# Fabrication of Zinc Oxide Nanoparticles Encapsulated Locust Bean Gum for Wound Healing: In Vitro/In Vivo and Molecular Docking Approach

**DOI:** 10.3390/ph19071015

**Published:** 2026-06-30

**Authors:** Sara Mehreen, Adeel Sattar, Faisal Usman, Muhammad Ovais Omer, Mian Abdul Hafeez

**Affiliations:** 1 Department of Pharmacology and Toxicology, Faculty of Bio-Sciences, University of Veterinary and Animal Sciences, Lahore 54000, Pakistandrovaisomer@uvas.edu.pk (M.O.O.); 2 Department of Pharmaceutics, Faculty of Pharmacy, Bahauddin Zakariya University, Multan 60800, Pakistan; faisal.usman@bzu.edu.pk; 3 Department of Parasitology, Faculty of Veterinary Sciences, University of Veterinary and Animal Sciences, Lahore 54000, Pakistan; abdul.hafeez@uvas.edu.pk

**Keywords:** zinc oxide nanoparticles, locust bean gum, hydrogel, wound healing, molecular docking

## Abstract

**Background**: Hydrogel membranes are highly effective biomaterials with huge potential for advanced wound management, offering the dual advantage of maintaining a beneficial moist environment while serving as a localized reservoir for antibacterial agents. Zinc oxide nanoparticles (ZnO NPs) are particularly notable in this regard, possessing potent antibacterial capabilities and intrinsic tissue-healing properties. **Methods**: In this study, we report the successful fabrication of a novel locust bean gum (LBG) hydrogel encapsulated with ZnO NPs, utilizing AlCl_3_ as a cross-linking agent. The synthesized nanocomposite hydrogels were structurally and chemically characterized using Scanning Electron Microscopy (SEM) and Fourier-Transform Infrared Spectroscopy (FTIR) followed by in vivo studies using experimental animals by creating wound model. **Results**: Physicochemical evaluations revealed a concentration and pH-dependent swelling profile, achieving a maximum swelling capacity of 97% at pH 9. In vitro kinetic studies depicted a highly desirable initial burst release of the active therapeutic, subsequently followed by a continuous, sustained release phase that was strictly governed by non-Fickian diffusion mechanics. Furthermore, the optimized formulations achieved excellent entrapment efficiencies (>95%) and substantial free-radical scavenging antioxidant potential (>86%). Biological assessments confirmed the safety and efficacy of the nanocomposites. The formulations exhibited zero cellular toxicity against fibroblast cell lines and demonstrated complete biocompatibility during tissue histopathological evaluations. Significant antimicrobial activity was also observed, as demonstrated by reduction in the Minimum Inhibitory Concentration (MIC) against critical pathogens, including *S. aureus*, *E. coli*, *P. aeruginosa*, and resistant MRSA strains. Crucially, in vivo studies using experimental animal models demonstrated accelerated tissue remodeling, achieving complete wound healing by day 11 and vastly outperforming the control groups. Finally, in silico molecular docking simulations corroborated these empirical findings, revealing strong and favorable binding interactions of the nanocomposite with key target proteins to elucidate its underlying antibacterial mechanisms. **Conclusions**: Collectively, these results establish the ZnO-loaded LBG hydrogel as a safe, multifunctional, and highly efficient topical drug delivery platform for cutaneous wound healing.

## 1. Introduction

The wound is a structural disruption in the skin resulting from physical trauma, injury or underlying pathological conditions potentially compromising local physiological stability and normal body functions. Wound healing is an intricate process that includes hemostasis, inflammation, proliferation and remodeling where disruptions impede tissue repair and lead to complications such as bacterial infections, fever and sepsis [1]. Neutrophils promote inflammation by releasing free radicals and inflammatory cytokines aiding in wound decontamination, followed by apoptosis and clearance by macrophages. Macrophages further eliminate bacteria and debris, preparing the wound for tissue regeneration [2].

Advancements in wound healing have been significantly influenced by nanotechnology, enhancing recovery in wound healing and microbial control. Metallic nanoparticles characterized by their economic feasibility and structural stability are of prime importance for therapeutic use [3]. These nanomaterials possess remarkable biological activity owing to their three-dimensional crystalline architecture and nanoscale dimensions ranging typically below 100 nm [4]. These particles exhibit enhanced surface area-to-volume ratio and adaptable physicochemical attributes. Such characteristics facilitate their use as drug carriers offering high stability, efficient drug loading, targeted delivery and controlled release dynamics. Metal nanoparticles contribute to wound healing primarily by lowering mitochondrial transmembrane potential, facilitating apoptosis of neutrophils, which results in subsequent decline in cytokine production [3]. The photothermal properties of metal nanoparticles promote the growth and movement of epidermal and fibroblast cell proliferation, fostering faster wound healing and bacterial eradication. Their antimicrobial function is driven by their ability to breach bacterial membranes, cause intracellular damage and stimulate the production of reactive oxygen species (ROS) [5].

Numerous studies have focused on biosynthesis of metallic nanoparticles including silver, gold, titanium dioxide, magnesium oxide, copper oxide, iron oxide, aluminum oxide and zinc oxide, revealing advancements in nanotechnology [6]. The effective use of coinage metal-based nanostructures in biomedical and other practical fields require their encapsulation within a natural or synthetic polymer framework, producing polymer/metal nanocomposites suitable for targeted applications.

Zinc oxide (ZnO) is being increasingly explored particularly as a multifunctional nanomaterial demonstrating its efficacy in antimicrobial therapies, cancer treatment, diabetes management, inflammation modulation and biosensing technologies. ZnO NPs are distinguished by their exceptional stability, non-toxicity and ease of fabrication. ZnO NPs have been designated by the FDA-USA as the safest nanomaterials, making them ideal for therapeutic and biomedical use [7,8].

Multiple studies have confirmed that ZnO NPs cause minimal harm to human cells while exhibiting significant antibacterial activity. Additional toxicity evaluations have shown that Zinc ions do not induce DNA damage in human cells thus confirming the nanoparticles biocompatibility [9]. The antimicrobial potential of ZnO against a variety of pathogens including *Bacillus subtilis*, *Staphylococcus aureus*, *Pseudomonas aeruginosa*, *Escherichia coli*, *Candida albicans* and *Aspergillus niger* is well established [10,11]. Moreover, Zinc plays a critical role in cell growth and proliferation, with elevated concentrations observed in wound tissue relative to normal tissue; insufficient Zinc levels have been linked to delayed healing. The presence of ZnO NPs further improves wound recovery by supporting fibroblast adhesion, encouraging keratinocyte migration and facilitating re-epithelization, along with their inherent anti-inflammatory and antiseptic characteristics [12].

Natural gums are ideal candidates for fabrication of hydrogels owing to renewable nature and presence of abundant hydrophilic moieties. The formation of hydrogen bonds along with other interactions such as cross-linking, electrostatic and hydrophobic forces underpins the development of hydrogels with excellent mechanical strength, thermal stability, and favorable rheological behavior. Moreover, natural gums can be chemically tailored to optimize hydrogel properties, with their performance influenced by their surface charge and structural configuration [13].

Locust bean gum (LBG) is a plant-derived polysaccharide with a molecular architecture consisting of 1,4-linked β-D-mannopyranosyl units with 1,6 α-D-galactopyranosyl side chains. Its exceptional biocompatibility, non-toxic nature, biodegradability render’s LBG and its derivatives highly attractive for biomedical and pharmaceutical applications. LBG offers considerable viscoelasticity and the ability to retard release, critical for achieving sustained therapeutic effects. By integrating other polymers through grafting, one can optimize the physicochemical properties of LBG-based hydrogels without compromising their inherent non-mutagenic and biocompatible attributes [14].

Hydrogels, in particular, offer the dual benefit of an effective protective barrier coupled with controlled permeability, thereby optimizing the wound environment for faster healing. Their adaptability to different wound geometries promotes optimal hydration, facilitates exudate removal and tissue repair [15,16]. Although conventional wound dressings are cost-effective and simple to use, but their limited efficacy, weak hemostatic function and adhesion to granulation tissue may compromise the process of wound repair [17]. There is a growing demand for novel dressings that not only control bleeding and manage exudate but also inhibit infections and encourage tissue regeneration. Ideally, a dressing must be biocompatible, serving as a protective barrier against pathogens besides enabling gas exchange to preserve wound hydration and remove excess fluid [18]. In pursuit of these clinical requirements, advanced dressings such as hydrogels, foams, gauzes, hydrocolloids, and polymeric films have been developed.

It is evident that wound healing capabilities of ZnO NPs can be customized by fabricating a drug delivery platform. Therefore, we intend to develop LBG hydrogel containing ZnO NPs as active constituent for evaluation of its wound healing abilities.

## 2. Results and Discussion

### 2.1. LBG-Loaded Hydrogels Exhibit Strong Mechanical Strength and Physical Stability

The fabricated LBG-loaded hydrogels were smooth, thin, flexible with transparent texture and easily peelable. They were easily removed from the Petri dishes without the need of force and no signs of air bubbles were present in the fabricated hydrogels [19]. Various physicochemical properties of prepared hydrogels were evaluated besides visual investigations to verify appropriateness regarding handling, transportation as well as storage and the uniformity of the contents during the process of fabrication. The determined parameters included weight variation, uniformity of ZnO, thickness and folding endurance. Table 1 shows the selected formulations based on physical stability, no color change and uniformity of shape. Weight variation in the prepared hydrogels was in range of 0.08 ± 0.04 to 0.746 ± 0.08 (g) whereas thickness of the hydrogels ranged 0.017 ± 0.01 to 0.055 ± 0.02. These measurements were indicative of uniformity of the contents (both ZnO and formulation components) as increased concentration of LBG was associated with increased thickness and weight variation. Folding endurance of hydrogel indicates physical strength as well as elasticity which are crucial parameters to maintain the film integrity upon application to the wounds for obtaining adequate release of the drug at target site followed by optimal healing of the wounds. Folding endurance plays critical role in preventing breaking of the hydrogel during application besides keeping it intact at the right place. Folding endurance of all the hydrogels was more than 250-fold which indicates strong mechanical strength of the prepared LBG-loaded hydrogels.

### 2.2. Hydrogels Demonstrate Maximum Swelling Capacity in Basic Environments

Hydrogels exhibit continuous swelling due to persistent fluid uptake from the tissue until equilibrium is reached. The novel hydrogel formulations preserve its swollen morphology under physical stress and exhibit robust structural integrity. Its higher water retention efficiency supports long term storage in low-humidity environments [20]. Swelling property of hydrogel is controlled by inter-polymeric network (IPN) of gum and drug with aqueous media. Swelling of LBG-loaded hydrogel was observed at acidic, neutral, and basic (3, 7, and 9 respectively) pH as given in Table 2. Continual changes occurred in the IPN of hydrogel during drug delivery and it acts as hydrophilic barrier controlling water penetration and drug diffusion [21]. Highest percentage swelling was observed at basic pH owing to hydrolysis of carboxyl present at mannose chain, which is converted into carboxylate ion and results in ionic repulsion that increases the swelling property of LBG hydrogel. Whereas, swelling decreases in acidic media due to screening effect of counter ion that prevent effective repulsion between ions. The decreased swelling in acidic media is also attributed to protonation of carbonyl groups with a consequent decrease in anionic repulsive forces. At a lower pH, the ionization of hydroxyl and carboxyl groups of LBG is repressed by H ions with a decreased swelling. Cross-linking of polymeric network also has significant effect on swelling behavior [22]. Non-crosslinked hydrogel formulation LBG9 did not swell properly and disintegrated after 10 h. A lower concentration of cross-linking created appropriate spaces and voids in polymeric network that supports significant swelling, drug entrapment and release of drug, as compared to higher concentrations [23]. At higher concentration of cross-linking agent, the polymeric network becomes so compact that aqueous media could not penetrate easily into hydrogel network and swelling decreased effectively.

### 2.3. Formulations Achieve High Zinc Oxide Entrapment Efficiency up to 95.4%

**Figure 1:**
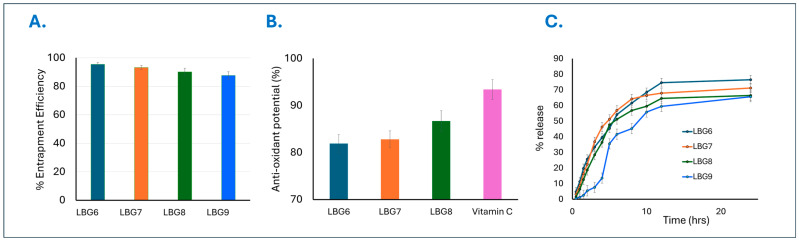
In vitro functional performance and release kinetics of the ZnO-loaded LBG hydrogels. (**A**) Entrapment efficiency across different formulations, demonstrating optimal encapsulation (>95%). (**B**) Antioxidant potential of the optimized hydrogels (LBG6, LBG7, and LBG8) compared to a Vitamin C control, showing significant free-radical scavenging capacity. (**C**) In vitro cumulative release profile of ZnO nanoparticles in a pH 7.4 phosphate buffer, exhibiting an initial therapeutic burst release followed by a sustained, non-Fickian diffusion pattern. *(Data represented as Mean ± SD).*

The entrapment efficiency of prepared hydrogel spanned from 87.6% to 95.4%. as depicted in Figure 1A. The efficiency was largely governed by polymer-to-gum ratio and the extent of cross-linker incorporation [23].

A series of 20 formulations incorporating various levels of locust bean gum and AlCl_3_ were systemically evaluated and the LBG6 variant featured more drug entrapment with least cross-linker requirement. Natural gum-based carriers incorporating metallic nanoparticles form hydrogels systems, where polymer swelling and cross-linked density enable stable encapsulation and controlled drug release. Matrix porosity and hydration levels significantly impact release rates [24]. The use of Al^3+^ ions as cross-linkers has been correlated with decreased entrapment efficacy likely due to water expulsion from tightly bound gel matrices [24]. As the cross-linker concentration increases, the percentage of drug encapsulation decreases. Correlating these findings with the SEM studies, it became evident that increased matrix porosity led to a proportional increase in drug entrapment. This trend was distinctly more pronounced at lower cross-linker concentrations, where expanded intermolecular spaces allowed a greater number of drug molecules to be effectively trapped. Conversely, elevating the cross-linker concentration formed a highly compact and tightly bound polymeric network, which restricted these internal voids and consequently reduced the overall drug entrapment [25].

### 2.4. ZnO-Loaded Hydrogels Significantly Reduce Inhibitory Concentrations Against MRSA and Other Pathogens

The synthesized ZnO NPs were found to exhibit antibacterial activity, with MICs determined as 32 µg/mL for *S.aureus*, 64 µg/mL for *E.coli*, and 128 µg/mL for *P. aeruginosa* [26]. ZnO NPs are reported to exhibit dose dependent antibacterial activity against *Staphylococcus aureus*, encompassing MRSA and MSSA isolates, with MIC ranging from 625 µg/mL to 5000 µg/mL, reflecting strain-specific resistance dynamics [27]. The incorporation of ZnO NPs into the hydrogel matrix resulted in strong activity against MRSA, as indicated by MIC and MBC values of 32 µg/mL and 64 µg/mL, respectively, highlighting its capacity to suppress resistant organisms [28]. It has been reported that co-administration of ZnO NPs with colistin, ciprofloxacin or meropenem against *P. aeruginosa* demonstrated enhanced efficacy and reduced MIC values [29]. Encapsulation of ZnO NPs in polymeric hydrogel formulation demonstrated superior antibacterial efficacy with reduced cytotoxicity compared to free ZnO NPs [19]. Similar results have been reported for *Moringa oleifra* gum-stabilized ZnO NPs, showing enhanced antibacterial activity against MRSA owing to lower MIC thresholds and improved bactericidal efficiency [30]. Metal nanoparticles exert potent antibacterial effects by destabilizing microbial membrane, initiating ROS mediated cytotoxicity pathways and interacting with bacterial genetic material, thereby disrupting cellular functions. These features enable their action against drug resistant pathogens [31].

Enhanced antibacterial activity was reported when silver nanoparticles were incorporated into locust bean gum/polyvinyl alcohol hydrogels, with MRSA inhibited at an MIC of 32 µg/mL. Activity of ZnO NPs hydrogels against *S. aureus*, *E. coli*, and *P. aeruginosa* was also demonstrated, with MICs of 16, 64, and 128 µg/mL, respectively, confirming the hydrogel’s broad spectrum antibacterial potential [32]. Antibacterial activity against MRSA strains showed significant results with decreased MIC, by ZnO-loaded LBGs optimized formulations LBG6, LBG7 and LBG8 with values ranging from 32 to 64 µg/mL, as depicted in Table 3. It has been reported that ZnO NPs accelerate the healing of MRSA infected wounds in rabbits. Similarly, ZnO NPs have been observed in bringing resistance modulation to MRSA and VRE strains sing quaternary ammonium compound coupled with LBG hydrogels. The LBG-loaded ZnO NPs hydrogel formulations (LBG6-LBG8) demonstrated MIC values ranging from 32 to 64 µg/mL against MRSA, in contrast to the higher values reported for ZnO NPs alone (256 µg/mL). These findings validated the synergistic interaction between LBG and ZnO, confirming the relevance of our formulation in wound care and resistance modulation.

### 2.5. Hydrogels Display Strong Antioxidant Potential and Effective ROS Scavenging Capacity

The accumulation of reactive oxygen species at wound margins can induce oxidative stress and initiate lipid peroxidation and may impede wound healing process. The incorporation of antioxidant agents has been proposed to promote tissue regeneration process by free-radical scavenging mechanism. Evidence from lead oxide-mediated skin toxicity model suggests that ZnO NPs exert potential antioxidant effects by reducing oxidative stress markers [33]. Mechanistic evaluation of LBG confirmed modest antioxidant potential, dominated by its ferrous ion chelation ability that impedes Fenton-driven oxidative damage [34]. Additional contributions from free-radical scavenging, particularly demonstrated by DPPH assay results and electron-donating activity reinforce its minimal but supportive redox regulatory influence. Its capacity to bind bile acids reinforces its applicability in systemic oxidative stress management through metabolic modulation [35]. The gum-based matrix served as a dynamic platform for stabilizing gold nanoparticles enabling improved antioxidant activity due to increased surface exposure and nanoparticle stabilization compared to nanocomponent system [36]. The antioxidant potential for LBG6, LBG7, LBG8 came out to be 81.9%, 82.8%, 86.7%, respectively with a control of Vitamin C showing 93.4% result as depicted in Figure 1B.

### 2.6. In Vitro Profiles Reveal an Initial Burst Followed by Sustained, Non-Fickian Drug Release

In vitro release studies of ZnO NPs across a cellophane membrane revealed an initial burst release, with over 50% of the incorporated ZnO NPs released within the first 6 h for most formulations, except for LBG9, which exhibited a release of over 40%. This initial burst was subsequently followed by a sustained release phase, as depicted in Figure 1C. This rapid initial release is primarily driven by the diffusion of active constituents located on the surface of the LBG films. During the drying process of the hydrogels, molecules typically migrate from the bulk matrix to the surface, resulting in rapid diffusion upon initial contact with the dissolution medium.

Achieving this high initial release of ZnO NPs is highly desirable at the wound site, as it rapidly establishes the required therapeutic effect. This is immediately followed by a continuous, sustained release of the internally encapsulated ZnO NPs to maintain prolonged antibacterial and healing action. This sustained release phase reflects an increased diffusion distance within the hydrogel matrix, confirming the successful deep encapsulation of ZnO NPs.

To elucidate the underlying mechanisms, the release data was kinetically evaluated using various pharmacokinetic models, including Zero-order, First-order, Higuchi, Hixson–Crowell, and Korsmeyer–Peppas. The regression coefficients (R^2^) for these models were determined to identify the best-fit model and establish whether the release pattern followed Fickian or non-Fickian kinetics. As detailed in Table 4, the Hixson–Crowell model provided the best fit, demonstrating the maximum values across all formulations. Furthermore, the diffusion exponent derived from the Korsmeyer–Peppas model was less than 0.5 for all formulations, with the exception of LBG9. This confirms a non-Fickian release profile, indicating that diffusion is the fundamental mechanism governing drug delivery from the matrix. Consequently, upon application to the wound site, the LBG hydrogel swells by absorbing water and tissue exudate, thereby facilitating the steady and sustained release of its therapeutic contents.

### 2.7. FTIR Analysis Confirms Successful Non-Covalent Integration of ZnO NPs

**Figure 2:**
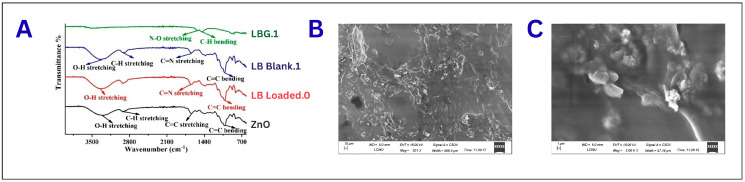
Structural evaluation of biomaterials. (**A**) FTIR spectra of the locust bean gum, blank film, ZnO-loaded hydrogel, and free ZnO nanoparticles. (**B**,**C**) SEM image of the ZnO-loaded hydrogels LBG7 and LBG8, respectively, illustrating the nanoscale spherical ZnO nanoparticles densely packed within the polymer matrix.

Spectral data from the FTIR analysis demonstrated that the ZnO nanoparticles (NPs) were successfully embedded within the LBG matrix, as evidenced by specific peak shifts and intensity changes relative to the blank sample. In FTIR analysis, LBG indicates pure LBG whereas LB blank shows control formulation without ZnO NPs. LBG loaded formulations indicated that ZnO NPs were encapsulated in the LBG hydrogels. As depicted in Figure 2A, the absence of any novel absorption bands suggests that the ZnO interacts with the polymer matrix entirely through non-covalent forces, such as hydrogen bonding or van der Waals interactions [7,37]. Furthermore, functional group analysis confirmed the presence of the LBG’s characteristic hydroxyl and ether moieties, while the emergence of distinct ZnO stretching bands below 900 cm^−1^ definitively validated the inclusion of the nanoparticles [38]. Notably, the retention of the hydroxyl groups (3200 cm^−1^ to 3550 cm^−1^) and the galactomannan-associated ether bands (1000 cm^−1^ to 1100 cm^−1^) within the spectra confirms the structural integrity of the LBG framework [39,40,41]. This preserved polymeric architecture directly correlates with the nanocomposite’s enhanced mechanical properties and its ability to facilitate sustained drug-release behavior in natural hydrogel systems (Table S1 entitled Spectral inferences of ZnO and locust bean gum loaded ZnO hydrogels).

### 2.8. SEM Imaging Reveals Dense Polymeric Packing and Uniform Nanoparticle Distribution

ZnO synthesis confirmation was observed through color change. The reaction mixture was light yellowish in color in the beginning followed by gradually changing to yellowish green indicating the formation of ZnO NPs. The surface morphologies of the synthesized ZnO nanoparticles (NPs), blank LBG hydrogel as depicted in Figure S1 (**SEM micrographs of zinc oxide nanoparticles (ZnO NPs) and locust bean gum (LBG) hydrogels)** and ZnO-loaded LBG hydrogel (Figure 2B,C) were evaluated using Scanning Electron Microscopy (SEM). The synthesized ZnO NPs exhibited a nanoscale, spherical morphology with a uniform distribution (see Figure S1 for comparison). A slight aggregation of the particles was observed, which is primarily attributed to the inherent polarity and electrostatic attraction of ZnO. The surface of the blank LBG hydrogel was dense, compact, and partially smooth, indicating good homogeneity and structural compatibility of the LBG polymeric matrix, which matches the FTIR results. Upon encapsulation of the ZnO NPs, the hydrogel matrix demonstrated an even more condensed packing structure. This enhanced structural density occurs because the nanoparticles, possessing a narrow size distribution, effectively occupy and fill the interstitial voids within the polymeric network alongside other fine particles.

ZnO NPs were further characterized by dynamic light scattering using Zeta sizer to corelate the dimensions as obtained from SEM analysis. The average particle size of ZnO NPs was 125.8 nm whereas polydispersity index (PDI) was 0.36 that indicates moderate uniform size distribution being consistent with slight particle aggregation in SEM analysis. Hydrodynamic diameter measured using DLS indicates solvated particles including the capping layer, surface hydration and moderate aggregation.

### 2.9. Hydrogels Accelerate Complete In Vivo Wound Closure Without Skin Irritation

**Figure 3:**
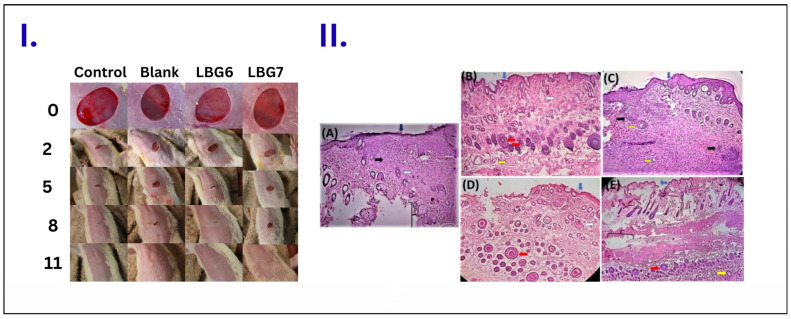
In vivo evaluation of full-thickness wound healing and tissue remodeling in Wistar rat models. (**I**): Macroscopic evaluation over a 12-day period. Representative images compare the progression of wound contraction in the control formulation (commercial Quench cream ^®^), blank LBG hydrogel (used as negative control) and optimized ZnO-loaded LBG hydrogels LBG6 and LBG7 at days 0, 2, 5, 8, and 11. (**II**): Histopathological evaluation. Images depict the untreated Control (**A**), showing high levels of inflammatory cells, edema, and dilated blood vessels. In contrast, the ZnO-loaded hydrogels exhibit accelerated healing: LBG6 (**B**) displays marked hair follicles, sebaceous glands, and comparatively minimal structural change; LBG7 (**C**) shows epidermal folding with focal dermal thickening; along with LBG8 (**D**) and the commercial Market formulation Quench cream^®^) (**E**). All LBG formulations achieved complete re-epithelization. The blue arrows represent dilated blood vessels, the black arrows point to hair follicles, the red arrows highlight sebaceous glands, and the yellow arrows indicate areas of dermal thickening.

Compared to conventional ZnO particles, ZnO NPs achieved superior wound healing requiring half of the MIC of standard ZnO particles, lowering the effective dose and mitigating potential toxicity. Safety evaluations, including 14-day corrosion and acute dermal irritation studies, revealed no evidence of edema, erythema, or allergic responses following the application of both blank and ZnO-loaded LBG hydrogels. These findings establish the biocompatibility and safety of the formulations for topical wound management. Subsequently, the in vivo wound healing efficacy of the optimized LBG formulations was evaluated using full-thickness wound models in Wistar rats. The percentage of wound contraction was systematically measured and compared against untreated controls, the blank hydrogel, and commercial positive control (Figure 3). Notably, animals treated with the ZnO-loaded hydrogels exhibited significant wound contraction as early as day 4, visually outperforming the commercial market product. Complete healing and wound closure were achieved by day 11 using the optimized LBG7 and LBG8 formulations. We anticipate that the loaded hydrogels significantly accelerate recovery by creating a protective, microbe-free environment while suppressing reactive oxygen species (ROS) to optimal levels. Thus, the observed superior healing performance is driven by the potent antibacterial and anti-inflammatory properties of the nanocomposite matrix (see Figure 2).

### 2.10. Histological Assessments Show Complete Re-Epithelization and Accelerated Tissue Remodeling

All the animals treated groups displayed complete re-epithelization and a reduction in wound dimensions. However, variations were observed in scar formation and tissue remodeling. The control and blank groups also had many inflammatory cells, edema, and dilated blood vessels as evident in Figure 3 (II). However, the animal group treated with LBG6 loaded hydrogel showed a disrupted epidermis with underlying edema, sebaceous glands, and a marked number of hair follicles. The wounds treated with LBG7 showed folding in the epidermis, with focal areas of increased thickness observed in the dermis. A comparatively less structural change than others was observed in the LBG6 group. ZnO NPs also accelerated tissue remodeling through anti-inflammatory and self-healing properties, respectively. LBG6 expedited the wound healing process in comparison to all other formulations. Because cross-linking affects the release of ZnO from the hydrogel matrix, it compromises septic conditions and inflammatory reflexes, which leads to delayed wound healing. However, all hydrogel formulations showed accelerated healing against blank and control. It can be depicted that hydrogel releases the drugs into injured tissues in a sustained manner to promote tissue remodeling in the wound healing process.

### 2.11. Cytocompatibility Assays Confirm the Absence of Toxicity Towards Fibroblast Cell Lines

Complete safety of ZnO-loaded hydrogels is critical to promote wound healing effectively besides bacterial growth limiting behavior. Cell viability of ZnO NPs, blank LBG hydrogel and various formulations of ZnO-loaded LBG hydrogels depicted no toxicity to the fibroblast cells. % Viability of ZnO NPs in concentration of 0.02 g/mL was 95 ± 1.6% following 24 h study by MTT showing its safety for use in hydrogels for wound healing. A decreased cell viability was observed with increasing concentration of LBG owing to increased viscosity affecting cell growth as well as functions. Increased concentration results in more polymeric networks with reduced diffusion of cell nutrients resulting in decreased cell viability. The % viability was still more than 80% for LBG 9 that is evident of its use as effective wound dressing. Safety of ZnO NPs loaded LBG hydrogels results from the possible change in specific surface area of NPs in the presence of polymeric material resulting in change in uptake as well as dissolution behavior. Cytotoxicity of ZnO NPs is dependent on type of cell, concertation and exposure time. The results suggested that ZnO NPs loaded LBG can be used as an excellent wound healer with no cell toxicity.

### 2.12. Molecular Docking Reveals Strong Binding Affinities to Antibacterial and Wound Healing Targets

**Figure 4:**
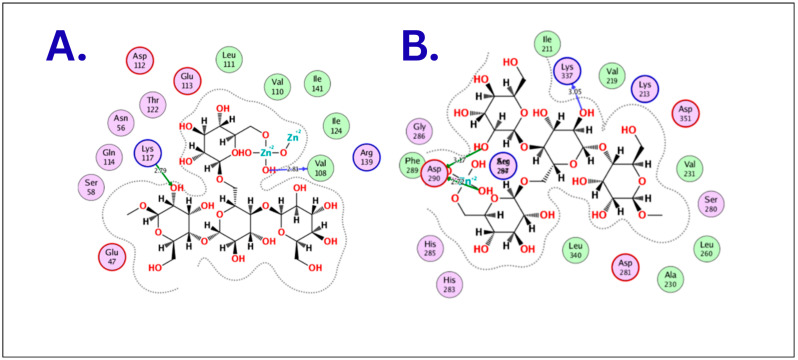
Molecular docking simulations illustrating the binding mechanisms of the LBG-ZnO ligand. (**A**) 2D interactions with the antibacterial target protein 2MLM (*S. aureus* sortase A). (**B**) 2D interactions with the wound healing target protein 6B8Y (TGF-β receptor). The molecular distances (Å) of prominent hydrogen bonds are indicated, confirming robust binding affinities with both therapeutic targets.

Inspired by the promising in vitro antibacterial and wound healing properties of the locust bean gum-loaded zinc oxide nanoparticles (LBG-ZnO), silico molecular docking studies were conducted to elucidate their suggestive mechanisms of action at the molecular level (Figure 4). The test compound was successfully docked into the three-dimensional structures of the target proteins, 2MLM (S. aureus sortase A) and 6B8Y (TGF-β receptor). The simulations revealed highly favorable binding affinities and stable interaction profiles for LBG-ZnO across both targets (see Figures S2 and S3 entitled **2D and 3D molecular docking interactions of the LBG-ZnO nanocomposite and respective cognate (crystallized) ligands with antibacterial target (*Staphylococcus aureus* sortase A; PDB ID: 2MLM) and 2D and 3D molecular docking interactions of the LBG-ZnO nanocomposite and respective cognate (crystallized) ligands and Wound-Healing Target (Transforming growth factor-beta (TGF-β) receptor; PDB ID: 6B8Y) respectively** for details).

Specifically, LBG-ZnO exhibited a favorable predicted binding pose score of −6.0 kcal/mol against the antibacterial target 2MLM. As illustrated in Figure 4, the nanocomposite established robust hydrogen bonds with key amino acid residues VAL108 and LYS117, at distances of 2.81–2.83 Å and 2.79 Å, respectively. These stable interactions underscore the significant antibacterial potential of the LBG-ZnO formulation.

Furthermore, Figure 4 depicts the interactions between the test compound and the wound healing transforming growth factor-beta (TGF-β) receptor (PDB ID: 6B8Y). The LBG-ZnO nanoparticles established a substantial hydrogen-bonding network within the active binding pocket, interacting directly with residues LYS232, ASP290, and LYS337. These robust intermolecular interactions culminated in a remarkable binding affinity, showcasing a docking score of −6.3 kcal/mol. Comprehensive details regarding the binding affinities, RMSD refine values, and molecular interactions with key amino acid residues for the LBG-ZnO test compound are summarized in Table S2 depicting Molecular docking score, RMSD refine, Hydrogen bonding, and ionic interactions with distances (Å) for studied compounds with target proteins including 2MLM and 6B8Y.

### 2.13. Bioadhesion Studies Demonstrate Enhanced Mucin Interactions for Optimal Topical Delivery

The bioadhesive strength of ZnO NPs loaded in LBG hydrogel was quantified using tensile detachment testing against mucin substrates, with F_max_ and W from work-distance profiles. LBG films alone yielded detachment forces of 0.25 to 0.4 N and adhesion work of 25–40 mJ, reflecting moderate adhesion [42]. ZnO NPs alone showed weak mucin binding and improved mucoadhesive property is obtained once they are incorporated into polysaccharide carrier like locust bean gum or chitosan [43]. Incorporation of ZnO into LBG matrices enhanced adhesion significantly, with Fmax values of 0.45–0.6 N and W values of 50–70 mJ, highlighting synergistic hydrogen bonding and electrostatic interactions with mucin. The strengthening of mucin interactions in ZnO-LBG hydrogel has been attributed to synergistic mechanisms involving hydrogen bonding, electrostatic attraction, and polymer chain entanglement. As a result, higher detachment forces and adhesion work have been compared to their either component alone, making these hydrogels suitable for topical drug delivery [44].

## 3. Materials and Methods

Zinc oxide nanoparticles were synthesized in house by method reported in Section 3.2. Locust bean gum was purchased from Krungthechemi, Bangkok, Thailand. Aluminum chloride (AlCl_3_), Tween 80 were obtained from Duksan (Pvt) Ltd. Daejeon, Republic of Korea. Double distilled water was obtained from Department of Pharmaceutics, Bahauddin Zakariya University Multan, Pakistan. All the chemicals were of analytical grade and used without any further modification. All experiments were performed in triplicate and the results are expressed as mean ± standard deviation (SD).

### 3.1. Preparation of Hydrogels Encapsulated ZnO NPs

LBG solution of various concentrations (1%, 3%, 5% *w*/*v*) was prepared at pH 7. After homogeneous mixing of AlCl_3_ solution, 0.8% *w*/*v* tween was added with continuous agitation. Upon complete mixing, 0.06% *w*/*w* ZnO NPs were added with constant stirring until uniformly dissolved followed by pouring onto Petri plates and kept in oven for 24 h at 60 °C and preserved for further use. A total of 20 different formulations were prepared by changing concentrations of LBG and AlCl_3_ as given in Table 1.

### 3.2. Zinc Oxide Nanoparticles Synthesis

The precursor solution was synthesized by combining 5.48 g of Zn (CH_3_COO)_2_.2H_2_O and 10.0 g of NaOH in purified water, forming a 25 mL solution with [Zn^+2^] = 0.5 mol/L and [OH^−^] = 5.0 mol/L. A mixture comprising 4 mL of precursor solution and 44 mL of absolute ethanol was stirred continuously in a beaker. Before autoclaving the mixture was subjected to ultrasonic treatment for 30 min. The temperature of autoclave was elevated to 110 °C and maintained for 13 h before gradually cooled to ambient conditions. The final product was rinsed thrice using absolute alcohol and deionized water followed by vacuum drying at 60 °C [45]. Particle size was determined by dynamic light scattering using zeta sizer by dispersing the NPs in suitable solvent followed by sonication to ensure homogeneity and measurements were carried out at 25 °C.

### 3.3. Swelling Indexo

The ability of hydrogel to retain moisture and absorb exudate is quantified by swelling index. Pre-weighed hydrogels (blank and loaded) were immersed in freshly prepared phosphate buffer (pH 7.4) and their weight measurements were taken at specific intervals and the swelling index was determined using the corresponding formula [46].

(1) $$\mathrm{Swelling}\;(\%)=\frac{Final\;weight\;of\;hydrogel\;-\;Initial\;weight\;of\;hydrogel}{Final\;weight\;of\;hydrogel}\times 100$$

### 3.4. Zinc Release Analysis Using Atomic Absorption Spectroscopy

The quantification of Zinc released from prepared hydrogels was conducted using atomic absorption spectrometer. ZnO NPs loaded hydrogels weighing 0.1 g were applied on cellophane membrane that was already soaked in phosphate buffer 7.4 for 24 h, mounted on Franz Diffusion Cell using phosphate buffer of pH 7.4 in receptor compartment. Each aliquot at specified interval was appropriately diluted followed by centrifugation at 10,000 rpm for 30 min and filtered prior to zinc quantification. Zinc concentration at various intervals were determined via calibration curve, and zinc quantification was done using a standard dilution-based formula [12]:

(2) $$\mathrm{Total}\;\mathrm{zinc}\;\mathrm{concentration}(\mathrm{mg}/L)\;=C_{zt}(1-df)$$

where $C_{zt}$ is concentration of zinc at time t and df is dilution factor.

### 3.5. Fourier-Transform Infrared Spectrophotometer (FTIR)

FTIR spectroscopy (BRUKER, Alpha, Platinum-ATR, Billerica, MA, USA) was employed to investigate the possibility of chemical interactions between locust bean gum, ZnO NPs, and ZnO NPs loaded hydrogel specially focusing on their cross-linking behavior. Spectral scanning was conducted within the range of 4000 cm^−1^ to 500 cm^−1^ [47].

### 3.6. Scanning Electron Microscopy

The morphological characteristics of the hydrogel were assessed using Scanning Electron Microscopy (SEM). Hydrogel samples were affixed to an aluminum support with a conductive adhesive. Prior to imaging, a gold-sputter coating was applied to enhance conductivity. Imaging was conducted under an optimized SEM voltage of 20 kV, using a Nova Nano SEM [48].

### 3.7. In Vitro Permeation Studies

The permeation efficiency of drug loaded hydrogel formulations was assessed via Franz Diffusion cell. The receptor chamber contained phosphate buffer of pH 7.4, maintaining a stable temperature of 37 ± 1 °C to simulate physiological conditions. Throughout the permeation studies, sink conditions remained effectively regulated. Mice’s abdominal skin of full thickness was carefully prepared by removing hair, dissecting tissue and saline cleansing. Hydrogel was affixed to the skin surface in predefined quantities. Receptor fluid samples were withdrawn at specific intervals and replenished using fresh buffer and aliquots were analyzed for drug content following appropriate dilution [49].

### 3.8. Release Kinetics of LBG-Loaded Hydrogels

Drug release from fabricated hydrogels was carried to determine drug diffusivity. Various mathematical models define release kinetics were applied on release date from hydrogels to elucidate mechanism of drug release.

Zero-order kinetics, in which release rate is independent of concentration, is determined by the following Equation:

(3) $$A{}^{\circ}-A_{t}=\mathrm{kt}$$

A° = Drug present in dosage form

A_t_ = Drug released at time t

K = Zero-order rate constant

The first-order kinetics, first introduced by Gibaldi and Feldman, explains concentration-dependent release, expressed by the following Equation:

(4) $$logA{}^{\circ}=logAt-\frac{k1t}{2.303}$$

K_1_ = First-order rate constant

The Hixon–Crowell model explains drug release based on dosage form dimensional changes, and is represented by the following Equation:

(5) $$A{}^{\circ}\frac{1}{3}-At\frac{1}{3}=k_{s}t$$

K_s_ = Hixon–Crowell constant

Higuchi’s model, developed in 1961, quantifies from porous matrices following a pseudo-steady-state approach, expressed by the following Equation:

(6) $$A=k_{H}t\frac{1}{2}$$

k_H_ = Higuchi’s constant

Korsmeyer–Peppas determines drug release from polymers following Fick’s law, as represented by the following Equation [50]:

(7) $$\frac{A_{t}}{A_{\infty }}\;=\;{kt}_{n}$$

*k* = Korsmeyer–Peppas constant

### 3.9. Antioxidant Study

A modified version of DPPH-based scavenging reaction mechanism was used to evaluate the antioxidant activity of the hydrogel samples. A volume of 100 uL of test solution was introduced into 3.9 mL solution of DPPH (0.1 mM, methanol-based), followed by 30 min of dark incubation. Absorbance at 319 nm was then recorded with UV-Visible spectrophotometer (UV-1206, SHIMADZU, Kyoto, Japan). The scavenging capacity was expressed as a percentage inhibition according to the following formula [51].

(8) $$\%\;\mathrm{Scavenging}\;\mathrm{activity}=\frac{{Absorbance}_{control}-{Absorbance}_{ZnO\;NPs}}{{Absorbance}_{control}}\times 100$$

### 3.10. Hydrogel Thickness and Folding Endurance

The mechanical integrity of the hydrogel was evaluated by continuous folding at a fixed position until breakage or 300 repetitions, reflecting acceptable structural integrity. Hydrogel thickness was evaluated through screw gauge measurements taken at six different sites, with the mean thickness value recorded for consistency

### 3.11. Tensile Strength

Specimens were prepared by cutting the dressings into a Dog-Bone shape (6 mm × 105 mm) with the aid of specially designed wooden blade while maintaining surface integrity. The tensile analysis was conducted with an Instron material testing machine with dressings symmetrically placed in the equipment’s grip [52]. Specimens were stretched at 300 mm/min until rupture, and tensile strength was computed as follows:

Tensile strength (kgf/cm^2^) = Max failure load(kgf)/Cross-sectional area (mm^2^) (9)

Elongation of the dressings was measured by calculating inter-clip distance changes as follows:

(10) $$\%\;Elongation=\frac{Final\;inter\;clip\;distance\;-\;Initial\;inter\;clip\;distance}{Initial\;inter\;clip\;distance}\times 100$$

### 3.12. Wound Healing Activity

Healthy adult male Wistar rats, weighing approximately 150 ± 25 g, were selected and monitored for several days prior to experimentation. Ethical approvals were granted by Ethical Review Committee of University of Veterinary and Animal Sciences, Lahore (NO. DR/312) and Pharmacy Ethical Committee, Bahauddin Zakariya University, Multan, Pakistan. Wound healing efficiency was examined using excision and burn wound models. Local anesthetic injection (Phenobarbital) was administered, and the hair from the dorsal pelvic area were removed before wound induction. Two wound models were employed: excision wounds were induced via scalpel blade removal of a 2 × 0.75 cm (1.5 cm^2^) skin section, and burns were created by a heating metal plate (1.5 cm ×1.5 cm). A total of 24 animals were used in the study and divided into four groups. The first group served as a control on which blank LBG hydrogel was applied, whereas the second group was given market formulation; two other groups were treated with LBG loaded with ZnO NPs formulations LBG 6 and LBG 7. The wounds were dressed using hydrogels, with daily bandage changes. Healing process was evaluated through wound contraction measurements, determined at 1–14-day intervals until full skin regeneration and calculated using Equation (11) by micrometer screw gauge [53]. The histopathology studies were done using hematoxylin eosin staining to study the changes at wound site and observe re-epithelialization.

(11) $$Wound\;contraction\%=\frac{{Area}_{day1}-{Area}_{day14}}{{Area}_{day1}}\times 100$$

### 3.13. Antibacterial Activity

The antibacterial effectiveness of hydrogel was determined via well diffusion method targeting Gram-positive (*Staphylococcus species*), Gram-negative (*Escherichia coli*, *Pseudomonas aeruginosa*) and MRSA strains. All the used strains were ATCC and obtained from the microbiology Laboratory of Department of Pharmaceutics, Faculty of Pharmacy Bahauddin Zakariya University, Multan, Pakistan. Bacterial suspensions were cultured in nutrient broth by a 24 h incubation at 37 °C. A 0.1 mL inoculum was evenly spread onto sterilized Petri plates under biological safety cabinets and wells were punched into the medium. The film blend solution was introduced at different concentrations followed by incubation at 37 °C for 24 h. Blank gel and a commercially available formulation Quench (Silver Sulphadiazine 1% *w*/*w*) for burn wound served as controls. The diameter of the inhibition zones was recorded using digital vernier caliper and compared with control measurements [54].

### 3.14. Bioadhesion Studies

Hydrogels were subjected to mucoadhesion analysis using a stable micro systems TA-XT2 Texture analyzer fitted with a 5 kg load cell. A rat small intestine model, thoroughly rinsed with saline, was utilized as a mucosal substrate to replicate wound-like adhesion conditions. Adhesion strength was measured by determining the maximum force (F_max_) required for film detachment, while the total adhesion work (W) was derived from the force–distance curve. Cohesiveness was characterized by measuring the displacement of film until detachment. The tests were conducted in triplicate to ensure reproducibility [55].

### 3.15. Weight Variation

Ten hydrogel samples, each precisely cut to a size of 1.5 cm × 1.5 cm, were weighed separately, and the data were presented as Mean ± standard deviation.

### 3.16. Cytotoxicity Studies

Hydrogels were assessed for cytotoxicity using MTT assay (3-(4,5-dimethylthiazol-2-yl)-2,5-diphenyltetrazolium bromide) on rat’s epithelial cells. Cells were first cultured in high-glucose DMEM (Dulbecco’s Modified Eagle Medium) enriched with 10% Fetal Bovine Serum (FBS), followed by incubation at 37 °C in a 5% CO_2_ environment for 24 h. The samples of the hydrogel preparations LBG6 to LBG9 (1 cm × 1 cm) underwent UV sterilization (30 min per side), and were rinsed thrice with sterile phosphate-buffered solution and DMEM. Epithelial cells were inoculated onto hydrogel-coated wells within a 24-well plate, while uncoated cells were designated as the negative control group. Incubation lasted for 24, 48, and 72 h. Each well received same volume of DMEM medium and MTT solution, and cells were incubated for additional 4 h at 37 °C. Formazan crystals were dissolved in 150 uL of DMSO, and absorbance at 570 nm was recorded and cell viability and determined using the following Equation [56].

(12) $$Cell\;viability\%=\frac{{{Absorbance}_{sample}-Absorbance}_{control}}{{Absorbance}_{control}}\times 100$$

### 3.17. Encapsulation Efficiency

To load the drug, pre-weighed sample was submerged in aqueous solution for 24 h. The sample was subsequently separated via decantation and subjected to hot-air drying at 50 °C until equilibrium was achieved [14]. Atomic Absorption Spectroscopy was employed to determine drug concentration in supernatant and the efficiency of drug loading was calculated using the following Equation:

(13) $$Encapsulation\;of\;drug(\%)=\frac{Final\;weight}{Initial\;weight}\times 100$$

### 3.18. Molecular Docking

The in silico study was conducted using MOE 2015.10 [57] to evaluate the binding affinity and orientation of zinc oxide nanoparticles of locust bean gum (LBG-ZnO) within the active sites of antibacterial target protein, such as sortase A from *Staphylococcus aureus* (PDB ID: 2MLM) [58,59] and wound healing transforming growth factor-beta (TGF-β) receptor (PDB ID: 6B8Y) [60]. The target protein 2MLM was resolved by NMR spectroscopy, demonstrating a significant percentile rank relative to all NMR structures, whereas the X-ray crystal structure of 6B8Y has a high resolution of 1.65 Å. The chemical structure of the test compound (LBG-ZnO) and crystallized ligands are drawn using ChemDraw Ultra 12.0 [6] and subsequently imported into MOE. Structural issues in the protein files were resolved using the QuickPrep module. Solvent molecules are removed, and hydrogen atoms are added to standard geometries. Energy minimization is performed on both the proteins and ligands using the MMFF94x force field. The prepared protein structures and ligands were saved in .mdb format for molecular docking simulations. The active sites of target proteins are defined as a region within 4.5 Å of the co-crystallized glycine ligand. The Dock module is used to perform docking of a test compound. The top five binding generated poses with the lowest binding energy and acceptable rmsd_refine values are selected for further consideration. The docking protocol is validated through redocking of the co-crystallized ligand into the active site of the target protein. The calculated RMSD value is less than 2 Å, confirming the reliability of the docking methodology.

## 4. Conclusions

The fabricated LBG-loaded hydrogels with encapsulated ZnO were of appropriate size, thickness, flexibility and homogeneity. Antioxidant and antibacterial properties were excellent with showing an increased trend with increasing the concentration of ZnO. Increased concentration of LBG resulted in denser and viscous polymeric network that sustained the release of ZnO with resultant biological effects. Significant reduction in MIC was observed against susceptible and MRSA strains. No evidence of chemical interaction was observed in FTIR Studies. Surface analysis showed smooth blank hydrogels with slight roughness on loading of ZnO whereas ZnO NPs were spherical shaped and agglomerated owing to polar interactions and size was in correlation with SEM analysis. Excellent swelling tendency was observed with the prepared hydrogel whereas the hydrogels were non-irritant and non-toxic to epithelial cell lines. In vivo wound healing in the rat models indicated faster wound healing in comparison to control exhibiting better epithelialization and tissues development. Molecular simulation revealed highly favorable binding affinities and stable interaction profile for ZnO and LBG across both targets. It is suggested that developed LBG-loaded ZnO NPs hydrogel can serve as promising candidate for wound dressing owing to better antibacterial, anti-inflammatory, antioxidant and wound healing properties besides non-toxicity. This can significantly reduce the cost at the end with improved compliance.

## Figures and Tables

**Table 1:** Formulation Composition and Physicochemical Parameters (Mean ± SD, *n* = 5).

Formulation Code	Locust Bean Gum (LBG) (% *w*/*v*)	Cross-Linker (AlCl_3_) (%)	Thickness (mm)	Folding Endurance	Weight Variation (g)
Blank Gel **	-	-	0.017 ± 0.01	292 ± 7	0.08 ± 0.04
LBG3	2	5	0.019 ± 0.02	294 ± 8	0.264 ± 0.03
LBG6	3	1	0.021 ± 0.02	312 ± 10	0.261 ± 0.02
LBG7	3	3	0.034 ± 0.01	296 ± 12	0.284 ± 0.03
LBG8	3	5	0.035 ± 0.02	304 ± 11	0.315 ± 0.05
LBG9	3	-	0.038 ± 0.01	319 ± 12	0.452 ± 0.04
LBG10 **	3	-	0.03 ± 0.01	295 ± 11	0.584 ± 0.07
LBG12	4	3	0.045 ± 0.02	305 ± 5	0.612 ± 0.09
LBG17	5	3	0.055 ± 0.02	317 ± 8	0.746 ± 0.08

** Indicates control/blank formulations where Zinc Oxide (ZnO) nanoparticles were omitted. All loaded formulations contain 0.06% *w*/*w* Zinc Oxide (ZnO) nanoparticles and 0.8% *w*/*v* Tween 80. Data represents mean ± standard deviation.

**Table 2:** Swelling Index (%) of LBG-loaded ZnO hydrogels (LBG6–LBG9) evaluated across time at acidic (pH 3), neutral (pH 7), and basic (pH 9) environments over 24 h.

Formulation	Time (hr)	Swelling at pH 3 (%)	Swelling at pH 7 (%)	Swelling at pH 9 (%)
LBG6	1	72	61	93
	2	74	62	94
	3	75	63	95
	4	77	65	95
	5	78	67	96
	6	79	68	97
	24	82	71	N/A
LBG7	1	65	51	80
	2	64	53	81
	3	67	54	83
	4	68	56	84
	5	69	57	85
	6	70	58	86
	24	73	63	N/A
LBG8	1	60	45	69
	2	61	47	69
	3	62	48	70
	4	64	48	71
	5	66	50	71
	6	67	51	72
	24	69	54	N/A
LBG9	1	51	40	64
	2	53	41	65
	3	54	42	66
	4	55	43	67
	5	55	45	68
	6	57	47	69
	24	57	50	N/A

**Table 3:** Minimum Inhibitory Concentration (MIC) values of Zinc Oxide-loaded locust bean hydrogels against various bacterial strains. All values are expressed in µg/mL and as mean ± SD, *n* = 5).

Formulation	*S. aureus*	*E. coli*	*P. aeruginosa*	MRSA
LBG (Blank)	--	--	--	--
ZnO NPs	32	64	128	256
LBG6	8	16	32	64
LBG7	8	32	64	128
LBG8	16	32	64	128
LBG9	32	64	128	256

**Table 4:** In vitro release kinetics and pharmacokinetic modeling parameters (Zero-order, First-order, Higuchi, Hixson–Crowell, and Korsmeyer–Peppas) of optimized LBG hydrogel formulations.

Formulation Code	Zero-Order Model		First-Order Model		Higuchi Model		Hixson–Crowell Model		Korsmeyer–Peppas Model		
	Ko	R^2^	K_1_	R^2^	K_H_	R^2^	K_HC_	R^2^	K_KP_	*n*	R^2^
LBG6	4.92	0.24	0.12	0.8	19.1	0.9	0.04	0.98	20.6	0.46	0.95
LBG7	4.78	0.78	0.12	0.9	18.8	0.9	0.04	0.95	21.7	0.43	0.84
LBG8	4.35	0.80	0.1	0.8	16.9	0.9	0.03	0.9	17.8	0.47	0.93
LBG9	3.81	0.86	0.07	0.8	13.8	0.9	0.02	0.9	9.3	0.67	0.92

## Data Availability

The data is contained within the article.

## Supplementary Materials

The following supporting information can be downloaded at: https://www.mdpi.com/article/10.3390/ph19071015/s1, Table S1: Spectral inferences of ZnO and locust bean gum loaded ZnO hydrogels. Table S2: Molecular docking score, RMSD refine, Hydrogen bonding, and ionic interactions with distances (Å) for studied compounds with target proteins including 2MLM and 6B8Y. Figure S1: SEM micrographs of zinc oxide nanoparticles (ZnO NPs) and locust bean gum (LBG) hydrogels. Figure S2: 2D and 3D molecular docking interactions of the LBG-ZnO nanocomposite and respective cognate (crystallized) ligands with antibacterial target (*Staphylococcus aureus* sortase A; PDB ID: 2MLM). Figure S3: 2D and 3D molecular docking interactions of the LBG-ZnO nanocomposite and respective cognate (crystallized) ligands and Wound-Healing Target (Transforming growth factor-beta (TGF-β) receptor; PDB ID: 6B8Y).
