# Supplementary material for: Fabrication of Zinc Oxide Nanoparticles Encapsulated Locust Bean Gum for Wound Healing: In Vitro/In Vivo and Molecular Docking Approach
Source: Pharmaceuticals (Basel). 2026 Jun 30;19(7):1015. doi: 10.3390/ph19071015 (PMC13415137; doi:10.3390/ph19071015)
Supplement: Supplementary file 1 [file pharmaceuticals-19-01015-s001.zip › pharmaceuticals-4330563-supplementary.pdf]

Table S1. Spectral inferences of ZnO and locust bean gum loaded ZnO hydrogels

Experimental Wavenumber(cm^{-1})	Vibrational Assignments	Calculated Wavenumber (cm^{-1})
3433.86, 3260, 3292	OH stretching	
2926.96, 2927	$-CH_2$ bending	
1000-1100,1008,1024	C-O-H stretching	1015.40
1306.87, 1374.92	$-CH_2$ stretching	1375.28
1626.42, 1634	C=O stretching	1636.84
400-800	ZnO stretching	654.48, 638.10

Table S2. Molecular docking score, RMSD refine, Hydrogen bonding, and ionic interactions with distances (Å) for studied compounds with target proteins including 2MLM and 6B8Y.

Compounds	Docking score (kcal/mol)	RMSD refine	H.B.I Residue (Distance Å)	Ionic & π-H bonding interaction
Target Receptor 2MLM				
LBG-ZnO	-6.0	2.2	VAL108 (2.81)<br>LYS117 (2.79)	--
CL	-4.9	1.8	ARG139 (2.90)	--
Target Receptor 6B8Y				
LBG-ZnO	-6.3	2.2	LYS337 (3.05)<br>ASP290 (3.17)<br>ASP290 (2.72)<br>LYS232 (2.78)	ASP290 (2.98)
CL	-6.9	2.4	--	LYS232 (3.61)

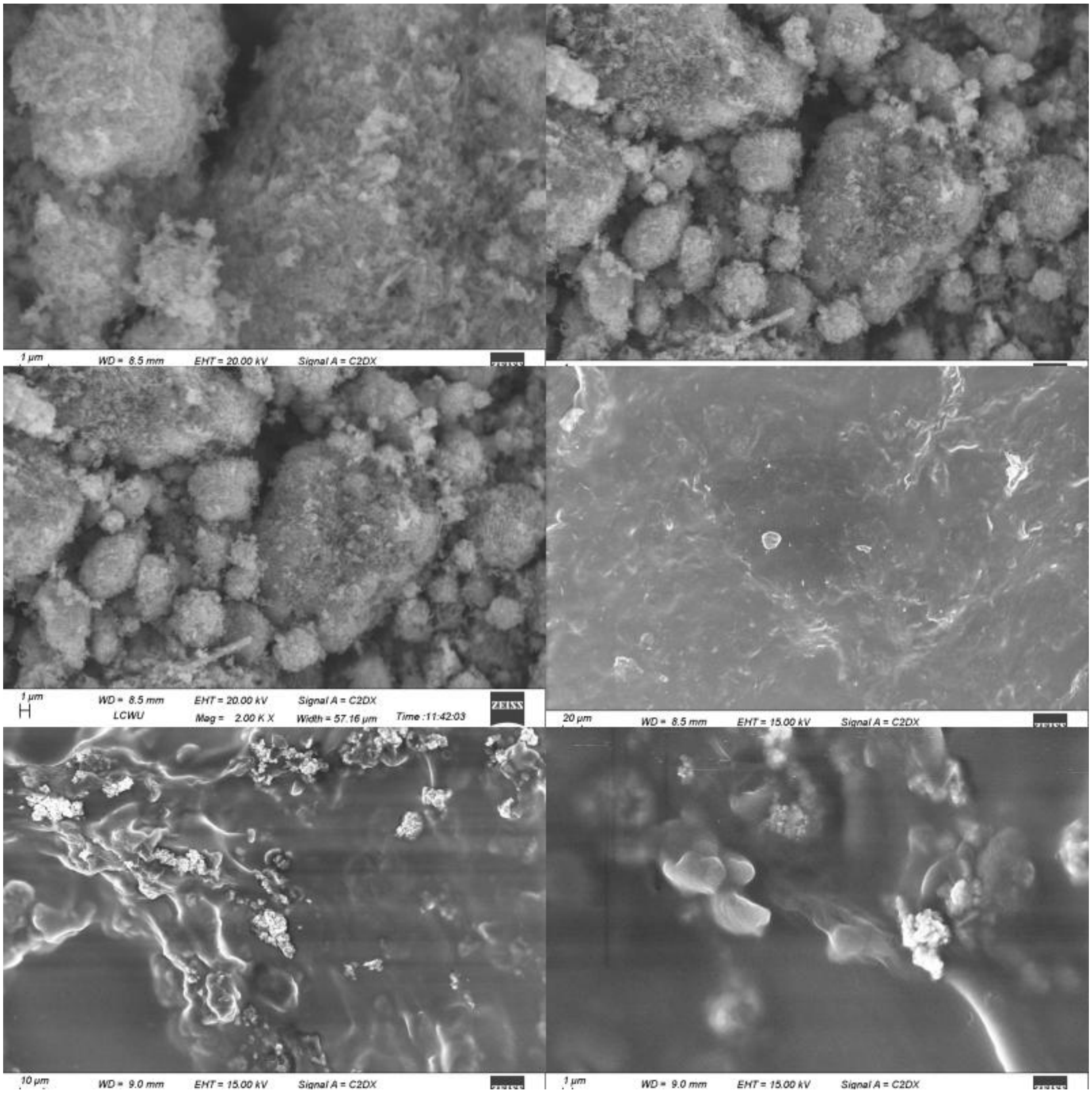

Figure S1. SEM micrographs of zinc oxide nanoparticles (ZnO NPs) and locust bean gum (LBG) hydrogels. (A–C) ZnO NPs showing spherical morphology with slight aggregation. (D) Blank LBG hydrogel exhibiting a smooth and dense structure. (E–F) ZnO-loaded LBG hydrogels (LBG7 and LBG8) displaying a more compact network with uniformly embedded nanoparticles, indicating successful encapsulation and enhanced matrix density.

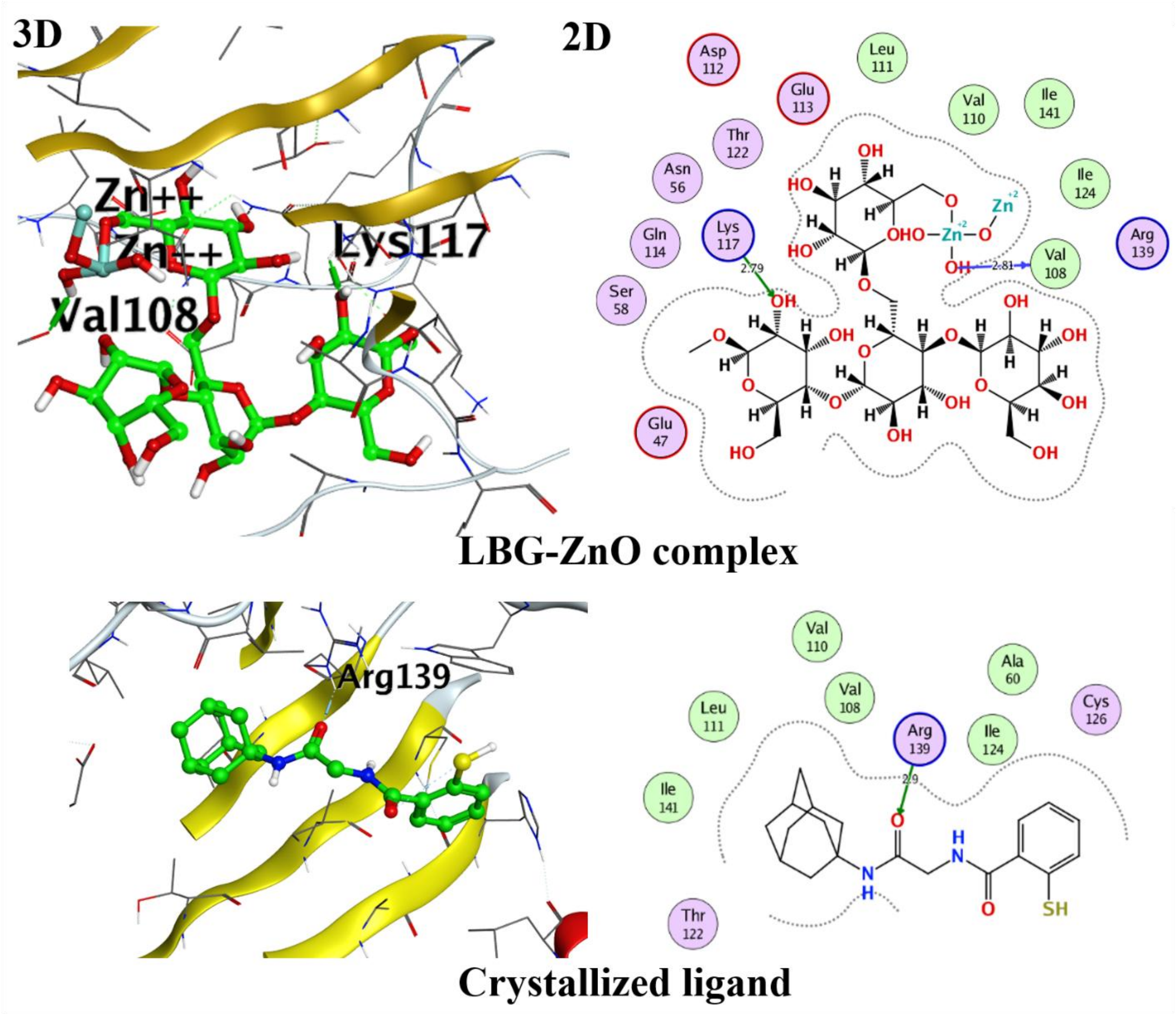

Figure S2. 2D and 3D molecular docking interactions of the LBG-ZnO nanocomposite and respective cognate (crystallized) ligands with antibacterial target (*Staphylococcus aureus* sortase A; PDB ID: 2MLM): Binding conformations within the active site demonstrate that the LBG-ZnO test compound establishes a strong binding affinity (docking score: -6.0 kcal/mol) through robust hydrogen bonding with key amino acid residues VAL108 (2.81 Å) and LYS117 (2.79 Å). In contrast, the cognate crystallized ligand (CL) exhibits a significantly weaker binding affinity (-4.9 kcal/mol), forming only a single hydrogen bond with residue ARG139 (2.90 Å)

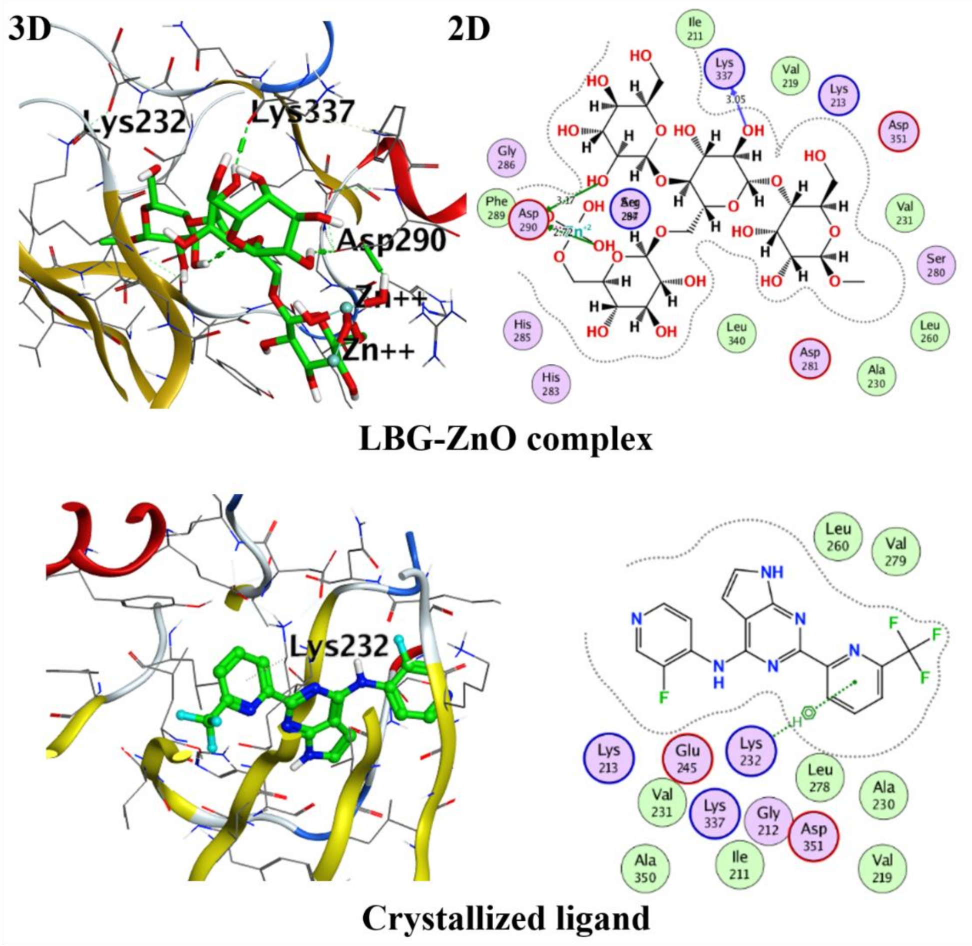

Figure S3. 2D and 3D molecular docking interactions of the LBG-ZnO nanocomposite and respective cognate (crystallized) ligands and Wound-Healing Target (Transforming growth factor-beta (TGF-β) receptor; PDB ID: 6B8Y): Binding orientations reveal that the LBG-ZnO nanocomposite successfully occupies the identical binding pocket as the cognate ligand. LBG-ZnO forms an extensive interaction network, engaging in hydrogen bonding with LYS337 (3.05 Å) and ASP290 (3.17 Å), alongside multiple ionic and π-H interactions with ASP290 (2.72 Å and 2.98 Å) and LYS232 (2.78 Å). These substantial interactions culminate in a highly favorable docking score of -6.3 kcal/mol. The cognate crystallized ligand for this target displayed a binding affinity of -6.9 kcal/mol, interacting primarily via hydrogen bonding with LYS232 (3.61 Å).
